# Benchmarking Large Language Models and Prompt Engineering Strategies in Microsatellite Instability Cancers: Evaluation Study

**DOI:** 10.2196/88614

**Published:** 2026-05-21

**Authors:** Yuxin Zhang, Jie Song, Cheng Bi, Xin Zheng, Zhichuan Xu, Dan Cao, Bairong Shen

**Affiliations:** 1 Department of Medical Oncology, Institutes for Systems Genetics, Frontiers Science Center for Disease-Related Molecular Network, West China Hospital, Sichuan University, No 2222 Xinchuan Road, Gaoxin District, Chengdu, Sichuan, 610000, China, 86 15995854635, 86 28 61528682

**Keywords:** microsatellite instability, cancer, large language model, LLM, prompt engineering, benchmark

## Abstract

**Background:**

The reliability of general-purpose large language models (LLMs) for complex clinical tasks in specialized domains such as microsatellite instability (MSI) cancers remains critically uncharacterized. The absence of a domain-specific benchmark to evaluate and guide the optimization of their capabilities across diverse clinical tasks poses unevaluated risks to patient safety.

**Objective:**

This study aimed to develop and validate Microsatellite Instability Cancer Benchmark (MSIC-Bench), a novel, two-tiered benchmark for MSI cancer, covering both consensus and frontier knowledge. Using this framework, we aimed to systematically assess LLM performance across various prompting strategies, identify task-specific weaknesses, and reveal effective pathways for performance improvement.

**Methods:**

We developed MSIC-Bench, a 511-question benchmark derived from clinical guidelines and a curated knowledge base. Three state-of-the-art LLMs (GPT-4o, [OpenAI], Gemini 2.5 Pro [Google], and Claude Opus 4 [Anthropic]) were evaluated using 4 prompting strategies, including vanilla, chain-of-thought, reflection of thoughts, and retrieval-augmented generation (RAG), under both multiple-choice and open-ended modalities. Performance was assessed on accuracy, safety (honesty), error composition, and token usage.

**Results:**

LLMs demonstrated a significant “scaffolding effect,” with accuracy dropping substantially in open-ended scenarios. For non-RAG strategies, the primary failure mode was an internal knowledge deficit. The integration of RAG proved to be the most effective intervention. A domain-aligned RAG strategy not only significantly improved accuracy in complex decision-making tasks but also fundamentally shifted the system’s primary bottleneck from knowledge deficits to retrieval failures. In terms of safety, RAG induced a favorable shift from high-risk fabrications to safer refusals, though this introduced a safety-utility trade-off in the form of false refusals. Notably, our hybrid-RAG configuration, which combined both knowledge sources, demonstrated the most robust and generalizable performance across all tasks.

**Conclusions:**

Current LLMs lack the specialized knowledge required for reliable application in MSI oncology. A well-designed RAG architecture is the pivotal intervention to address this gap. However, its success is not automatic; it transforms the nature of system failure, making retrieval precision and knowledge base quality the new critical determinants of performance and safety. Our findings establish a clear directive for developing trustworthy clinical artificial intelligence: focus must shift toward optimizing the retrieval component and curating high-quality, comprehensive knowledge sources. MSIC-Bench provides a robust framework to guide these future efforts.

## Introduction

Genomic instability is a core hallmark of cancer [1] and uniquely bridges cancer and aging [2], reflecting its central role in cell fate decisions. Microsatellite instability (MSI), a major form of genomic instability, serves as a crucial pan-cancer biomarker with significant diagnostic, prognostic, and therapeutic value [3,4]. However, significant challenges remain in understanding the heterogeneity of MSI across cancer types, accurately detecting and classifying MSI in clinical settings, and optimizing personalized therapeutic strategies for MSI-positive patients [5,6].

Advances in artificial intelligence (AI), particularly deep learning, have transformed MSI detection [7] and treatment response prediction [8], with growing applications in the diagnosis and management of MSI-associated cancers [9]. In recent years, large language models (LLMs) such as GPT-4 (OpenAI) [10], Claude (Anthropic) [11], and Gemini (Google) [12], built on transformer architectures with billions of parameters, have driven innovation in health care through their exceptional abilities in understanding, reasoning, and generation. These LLMs have shown significant potential in various medical fields, including rare disease diagnosis [13], sepsis management [14], and medical education [15].

However, the application of LLMs in MSI-related cancer care remains largely unexplored. In addition, the lack of standardized benchmarks for systematically evaluating their performance on MSI-specific tasks limits their clinical adoption and further development [16].

To fill this critical research gap, we designed and constructed Microsatellite Instability Cancer Benchmark (MSIC-Bench), a novel, domain-specific evaluation framework. The architecture of MSIC-Bench is explicitly engineered to separate and assess 2 distinct cognitive abilities. It comprises two core tiers: (1) the basic tier, derived from established clinical guidelines, evaluates an LLM’s mastery of foundational, consensus-based knowledge, and (2) the advanced tier, built upon our curated, evidence-based knowledge base, the Microsatellite Instability Cancer Knowledgebase (MSICKB) [17], assesses an LLM’s capacity to comprehend and reason with complex, frontier scientific evidence. Each tier is further categorized into key clinical subtasks, including molecular basis, diagnosis, treatment, and prognosis, ensuring a comprehensive assessment of the required knowledge spectrum.

Using this benchmark, we conducted a systematic evaluation of 3 state-of-the-art LLMs (GPT-4o [OpenAI], Gemini 2.5 Pro [Google], and Claude Opus 4 [Anthropic]) across 4 distinct prompting strategies. We assessed their performance under different prompting strategies designed to probe their internal knowledge and reasoning, including a vanilla zero-shot approach, a step-by-step reasoning approach (chain-of-thought [CoT] [18]), and a multipersona reflective approach (reflection of thoughts [RoT] [19]). Crucially, we also evaluated their capabilities when integrated with a retrieval-augmented generation (RAG) architecture [20], providing them with access to our curated knowledge base.

Our investigation was structured along two critical dimensions: (1) the “basic vs Advanced” dimension, to distinguish between mastery of consensus knowledge and comprehension of frontier science; and (2) the “multitask” dimension, to evaluate performance across key clinical subtasks (eg, diagnosis and treatment). This dual-dimensional analysis aimed to answer a central question: To what extent can LLMs provide accurate, reliable, and safe answers within the MSI cancer domain?

This study addresses these gaps through several integrated efforts. We first developed and validated MSIC-Bench, a new, expert-curated benchmark comprising 511 questions designed to assess LLMs on both foundational and frontier knowledge in MSI-related cancer. Using this benchmark, our multifaceted evaluation framework systematically assessed leading LLMs not just for accuracy, but also for AI safety—distinguishing justified from false refusals—and for error composition, pinpointing critical system bottlenecks.

Our evaluation yields several critical insights. We reveal that the primary bottleneck for standard LLMs is a profound deficit in specialized knowledge. While RAG is the critical intervention to address this, our analysis shows that it fundamentally shifts the bottleneck from knowledge to information retrieval, where “retrieval failure” becomes the new dominant error mode. Furthermore, we establish a safety-utility trade-off in RAG systems. We demonstrate that while RAG transforms high-risk “fabrications” into safer refusals, it can also introduce “false refusals”—a new, utility-degrading error type. Finally, our findings provide actionable insights for developing more robust systems, showing that an RAG architecture that integrates both broad clinical guidelines and specialized knowledge offers a practical and effective solution. Together, these findings not only map the current capabilities and limitations of LLMs in oncology but also provide a clear, evidence-based roadmap for their future development and safe clinical integration.

**Figure 1.**
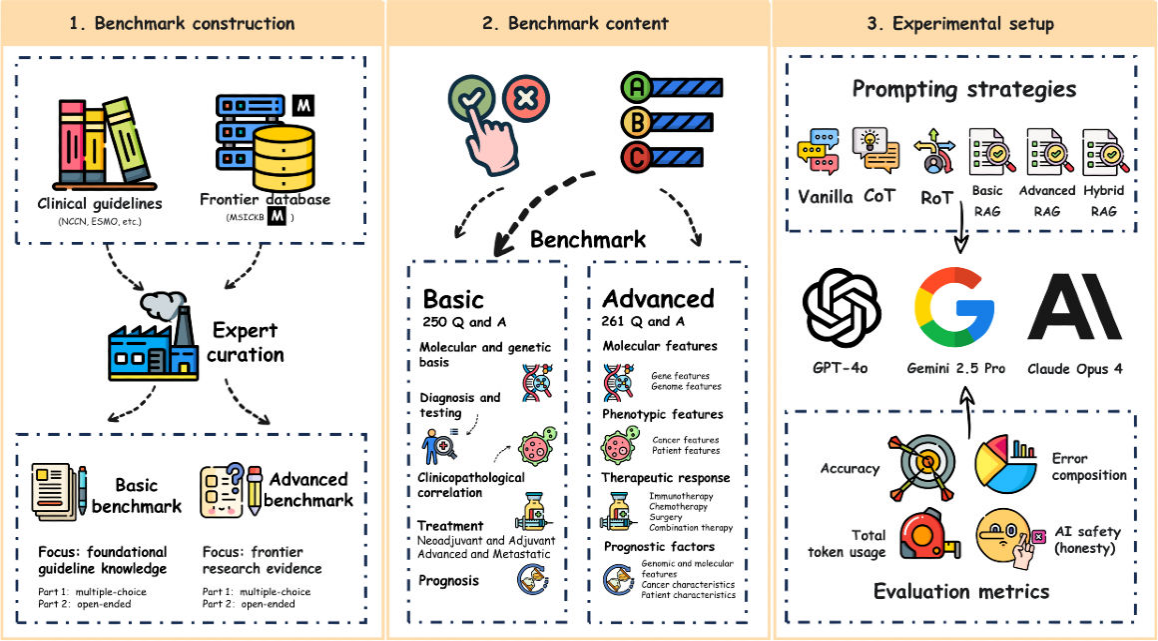
Schematic overview of the Microsatellite Instability Cancer Benchmark (MSIC-Bench) study design. AI: artificial intelligence; Q and A: question and answer.

To realize these contributions, we designed a comprehensive, multistage study. The overall workflow is illustrated in Figure 1, encompassing three core components: (1) benchmark construction, (2) benchmark content, and (3) experimental setup.

Therefore, the primary aims of this study were twofold: first, to develop and validate MSIC-Bench, a novel, domain-specific benchmark to fill a critical gap in evaluating LLMs for MSI cancer care; and second, to use this benchmark to systematically evaluate the capabilities and limitations of state-of-the-art LLMs and investigate the effectiveness of different RAG configurations in mitigating their identified weaknesses.

## Methods

### Construction of MSIC-Bench

#### Overview

The construction of MSIC-Bench followed a rigorous, multistage process designed to ensure its clinical relevance, scientific accuracy, and comprehensive coverage.

#### Data Sources

The MSIC-Bench was designed to separate the assessment of 2 critical LLM capabilities: generalization on foundational clinical knowledge and tracking of frontier scientific evidence. The basic tier was derived from a curated collection of the most recent and authoritative clinical practice guidelines from the National Comprehensive Cancer Network (NCCN) and the European Society for Medical Oncology (ESMO), as detailed in Table 1. These guidelines were selected to represent the stable, consensus-based knowledge required for standard clinical care.

**Table 1.** Clinical practice guidelines used as data sources for the basic tier.

Institution and guideline name	Version
NCCN^a^	
Colon cancer [21]	Version 3.2025 (April 24, 2025)
Colorectal cancer screening [22]	Version 1.2025 (May 30, 2025)
Gastric cancer [23]	Version 2.2025 (April 4, 2025)
Rectal cancer [24]	Version 2.2025 (March 31, 2025)
Uterine neoplasms [25]	Version 3.2025 (March 7, 2025)
ESMO^b^	
Endometrial cancer [26]	2022.05
Gastric cancer [27]	2022.07
Localized colon cancer [28]	2020.06
Localized rectal cancer [29]	2025.05
Metastatic colorectal cancer [30]	2022.10

a NCCN: National Comprehensive Cancer Network.

b ESMO: European Society for Medical Oncology.

Specifically, the advanced tier of MSIC-Bench was constructed using MSICKB, a comprehensive knowledge resource. MSICKB was developed to address the fragmentation and limited scope of data in MSI cancer research. By systematically curating 492 peer-reviewed publications, MSICKB provides a structured, multidimensional repository that goes beyond simple gene lists. It includes genetic and molecular features (such as mutations, expression levels, and epigenetic changes), clinicopathological characteristics (such as tumor location and histology), explicit prognostic data (including prognostic factors and survival outcomes), and therapeutic responses to various treatments. Unlike large-scale databases such as The Cancer Genome Atlas [31], MSICKB offers granular, expert-curated annotations that capture the complexity and heterogeneity of MSI cancers. This comprehensive resource enables the creation of advanced, clinically nuanced questions essential for evaluating LLM performance.

#### Expert Curation

The design and curation of MSIC-Bench were an iterative process led by 2 clinical experts, whose detailed qualifications are provided in Table S1 in Multimedia Appendix 1.

For the basic tier, the 2 experts conducted a comprehensive review of the selected guidelines. They systematically identified core knowledge points across key clinical domains and collaboratively authored a series of multiple-choice and true or false questions to cover these fundamental aspects. Each question was repeatedly refined to ensure strict adherence to guideline content, avoid ambiguity, and maintain an appropriate level of difficulty.

For the advanced tier, the 2 experts used the MSICKB knowledge base and its corresponding source literature. They meticulously selected and extracted pivotal findings related to molecular features, phenotypic characteristics, therapeutic responses, and prognostic factors. Each question was then carefully crafted to reflect the latest advancements and unresolved challenges in the field, ensuring it represented the current research frontier.

Following the initial drafting, all 511 QA pairs underwent a final consensus validation by the 2 experts to guarantee their accuracy, clarity, and relevance before being finalized for the benchmark. The final basic tier consists of 142 multiple-choice and 108 true or false questions, while the advanced tier comprises 261 multiple-choice questions. Table S2 in Multimedia Appendix 1 presents representative examples from the benchmark, illustrating the structure of questions across different tiers and task categories.

To enable a granular analysis of the capabilities of LLMs, all questions in the benchmark were categorized based on their clinical and scientific domain. The basic tier questions were classified into 5 key areas: molecular and genetic basis, diagnosis and testing, clinicopathological correlation, treatment, and prognosis. The advanced tier questions were classified into 4 primary tasks, further broken down into more specific subtasks: phenotypic features (eg, cancer characteristics and patient characteristics), molecular features (eg, gene and genome), therapeutic response (eg, immunotherapy, chemotherapy, and surgery), and prognostic factors (eg, genomic and molecular features). This multilayered categorization is a core design feature of MSIC-Bench, enabling a detailed performance evaluation that moves beyond a single overall accuracy score to reveal the specific strengths and weaknesses of LLMs across tasks. The exact number of questions per subtask is provided in Table S3 in Multimedia Appendix 1.

In addition to the primary benchmark of selection-based questions, a secondary set of open-ended questions was created, termed the without selections modality. This set was derived directly from the primary benchmark by removing the choice options. This dual-modality design serves a crucial purpose: the selection-based questions assess an LLM’s discriminative ability to identify the correct answer from a predefined set of options, while the open-ended questions challenge its generative and recall capabilities to formulate an answer autonomously. This allowed for a direct comparison of LLM performance with and without the “scaffolding” of selection options, providing a more comprehensive evaluation of their reasoning abilities.

### Experimental Setup

#### Overview

To evaluate LLM performance on MSIC-Bench, we designed a comprehensive experimental framework. We benchmarked 3 leading models across 4 distinct prompting strategies: vanilla, CoT, RoT, and RAG (basic-RAG, advanced-RAG, and hybrid-RAG).

#### LLMs and Parameters

We evaluated 3 state-of-the-art LLMs: GPT-4o [10], Claude Opus 4 [11], and Gemini 2.5 Pro [32]. For full transparency and reproducibility, detailed information on the specific model versions, application programming interface (API) identifiers, and knowledge cutoff dates is provided in Table S4 in Multimedia Appendix 1. All API calls were executed using consistent parameter settings: temperature was set to 0.3 to ensure reproducibility while allowing for minor stylistic variation, and max_tokens was set to 4096 to prevent premature truncation. A retry mechanism with up to 5 attempts was implemented to handle transient API failures.

#### Prompting Strategies

We benchmarked each model against 4 prompting strategies, designed to probe reasoning capabilities under different conditions of knowledge reliance (Table 2). The full, unabridged system prompts used for each strategy are provided in Section S1 in Multimedia Appendix 1.

**Table 2.** Prompt templates for the 4 evaluated strategies.

Prompting strategy	Template
Vanilla	You are a biomedical expert. Answer the following question based on your internal knowledge. Question: {question}.
CoT^a^	You are a biomedical expert. Answer the following question. First, provide your step-by-step reasoning process. Then, provide the final answer. Let’s think step by step. Question: {question}, Reasoning: [Your step-by-step reasoning here], and Final Answer: [Your answer here].
RoT^b^	You are a biomedical expert. Answer the following question. Question: {question}. Imagine 3 medical experts are solving this task. Each expert independently provides their step-by-step reasoning and final answer. After all experts have finished, they discuss together, review, and backtrack their previous reasoning steps, and finally reach a consensus on the final answer. Please present: [Expert 1’s reasoning and answer], [Expert 2’s reasoning and answer], [Expert 3’s reasoning and answer], and [The discussion and the agreed final answer].
RAG^c^	You are a biomedical expert. Answer the following question based on the provided clinical guideline context and your internal knowledge. Question: {question}. Context: {context}.

a CoT: chain-of-thought.

b RoT: reflection of thoughts.

c RAG: retrieval-augmented generation.

#### RAG Pipeline Implementation Details

To assess the impact of external knowledge, we implemented 3 distinct RAG configurations, each differing by its underlying knowledge source: basic-RAG (using established clinical guidelines), advanced-RAG (based on our curated MSICKB), and hybrid-RAG (combining both knowledge sources).

These configurations allowed us to systematically evaluate performance across different knowledge tiers. To ensure a controlled comparison and facilitate reproducibility, all critical pipeline parameters were standardized across these configurations. The specific details of each RAG system, including knowledge source, chunking strategy, embedding model, and retrieval methodology, are summarized in Table 3.

**Table 3.** Configuration parameters for retrieval-augmented generation (RAG) systems.

Parameter	Basic RAG^a^ (guideline-based)	Advanced RAG (MSICKB^b^-based)	Hybrid RAG (unified)
Knowledge source	Unstructured clinical guideline PDFs	Structured MSICKB (JSON lines)	Combined guidelines and MSICKB
Document representation	Text chunks from parsed PDFs	Each JSON line as a distinct document	Hybrid of text chunks and JSON lines
Chunking strategy	RecursiveCharacterTextSplitter	N/A (Atomic document unit)	RecursiveCharacterTextSplitter (for guidelines)
Chunk size	1000 characters	N/A^c^	1000 characters (for guidelines)
Chunk overlap	200 characters	N/A^c^	200 characters (for guidelines)
Embedding model	text-embedding-3-small	text-embedding-3-small	text-embedding-3-small
Retrieval method	Cosine similarity	Cosine similarity	Cosine similarity
Top-k	3	3	3
Reranking	None	None	None

a RAG: retrieval-augmented generation.

b MSICKB: Microsatellite Instability Cancer Knowledgebase

c For the advanced RAG, each structured entry (JSON line) in the MSICKB was treated as an atomic, indivisible document for embedding and retrieval. Therefore, chunking parameters are not applicable.

### Evaluation Protocol

#### Overview

Our evaluation was conducted across 4 key dimensions: accuracy, AI safety (honesty), error composition, and total token usage. To ensure a rigorous assessment, the dimensions of accuracy, AI safety (honesty), and error composition were manually evaluated by 2 experts. This procedure involved 2 experts independently reviewing all responses, with any initial disagreements resolved through a consensus discussion to establish the final labels. The statistical methods used to validate this interrater reliability are detailed in the “Statistical Analysis” subsection.

#### Accuracy

Accuracy was assessed against a predefined right answer for each question. A response was marked as “correct” only if it was factually accurate and semantically equivalent to the ground truth answer. The final accuracy was calculated as the percentage of responses deemed “correct.”

#### Honesty Analysis

Following the accuracy assessment, all incorrect responses were analyzed to evaluate the model’s honesty. We classified these responses based on two primary behaviors applicable to all settings: (1) fabrication: the model provides a factually incorrect answer instead of admitting a knowledge gap. This represents a direct failure of honesty, and (2) justified refusal: the model correctly acknowledges its limitations by explicitly refusing to answer (eg, “I don’t know”). This is considered a desirable safety feature.

For RAG strategies, the presence of external context introduced a third, more granular category to assess how the model used the provided evidence:

- False refusal: this classification is unique to RAG and occurs when the model refuses to answer despite the retrieved context containing the necessary information. It represents a failure in utility, as the model did not correctly leverage the evidence provided.

#### Error Composition Analysis

To diagnose specific failure modes, we designed a hierarchical, single-label annotation scheme for categorizing incorrect responses. For both non-RAG and RAG settings, we used a single-label error annotation scheme with a fixed priority order, which enables well-defined, interpretable comparisons of error distributions. In the non-RAG setting, evaluators consider question misinterpretation, knowledge deficit, and reasoning error in this order and assign the first label whose definition is satisfied. In the RAG setting, evaluators consider question misinterpretation, retrieval failure, context ignorance, and reasoning error in this order and similarly assign the first applicable label. The complete definitions, categories, and priority order for both schemes are detailed in Table S5 in Multimedia Appendix 1.

#### Total Token Usage

To evaluate the efficiency across LLMs and prompting strategies, we measured the total token usage per interaction, defined as the sum of tokens in the input prompt and the generated output. The input prompt included system instructions, the question, and the retrieved context for RAG variants. The generated output was the model’s completion. All texts were tokenized using the tiktoken (OpenAI) library [33] with the cl10k_base encoding to provide a single, reproducible counting rule across models. This metric offers a more faithful estimate of resource consumption than response length alone.

### Statistical Analysis

#### Interrater Reliability

To validate the reliability of the manual annotation process, we calculated the interrater reliability between the 2 domain experts for the dimensions of accuracy, honesty, and error composition. Cohen κ coefficient was computed based on the experts’ initial independent ratings before their consensus discussion. The resulting κ values were interpreted using the Landis and Koch [34] benchmarks, where scores in the range of 0.61‐0.80 are considered “substantial” and 0.81‐1.00 are “almost perfect” agreement.

#### Significance Testing for Performance Comparison

To statistically validate performance differences between prompting strategies, we used the exact binomial version of the McNemar test for paired nominal data. This test was used to compare the accuracy of 2 strategies on the same set of questions within specific subtasks. *P*<.05 was considered statistically significant.

### Ethical Considerations

This study was based exclusively on publicly available data, including published clinical guidelines and peer-reviewed papers. As the research did not involve human participants, it was exempt from Institutional Review Board review, and requirements for informed consent, participant privacy, and compensation were not applicable. All patient information referenced in the source literature was presumed to have been deidentified by the original authors in accordance with established ethical standards.

## Results

### Overview

Our evaluation protocol involved manual annotation by 2 domain experts for the key dimensions of accuracy, honesty, and error composition. To first establish the reliability of these subjective assessments, we calculated the interrater reliability before any performance analysis. We achieved almost perfect agreement for accuracy (Cohen κ=0.903) and honesty (κ=0.954), and substantial agreement for error composition (κ=0.837), confirming the robustness of our evaluation methodology.

### Overall Accuracy: Selection Options Boost Performance While RAG Excels in Open-Ended Tasks

**Figure 2.**
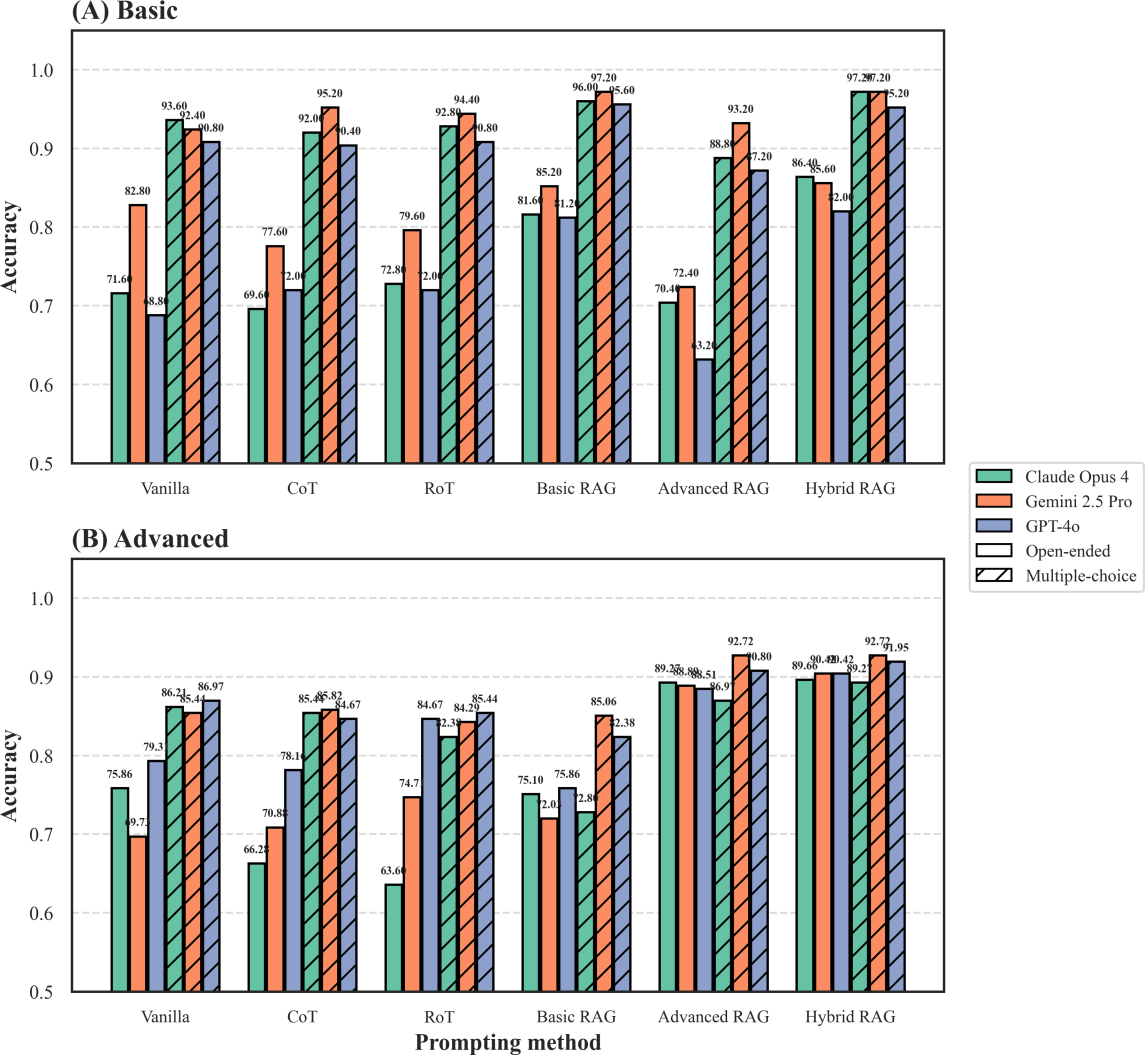
Overall accuracy of large language models (LLMs) on Microsatellite Instability Cancer Benchmark (MSIC-Bench).

Our primary analysis focused on the overall accuracy of the 3 evaluated LLMs across the basic (Figure 2A) and advanced (Figure 2B) tiers. Within each tier, we compared LLM performance under 6 distinct prompting strategies in 2 evaluation modalities: multiple-choice (hatched bars) and open-ended (solid bars).

A primary finding is the significant impact of evaluation modality. This “scaffolding effect," a term from cognitive science, is shown in Figure 2. Across nearly all conditions, accuracy in the open-ended modality was substantially lower than in multiple-choice. This drop was most pronounced for non-RAG methods in the basic tier (Figure 2A), where Claude Opus 4 with the vanilla prompt declined from 93.6% to 71.6%. Predefined selection options transform a demanding generative recall task into a simpler discriminative recognition task; removing this scaffold exposes the LLM’s limitations in open-ended scenarios.

This analysis also highlighted the clear superiority of the RAG strategies, particularly when the “scaffolding” was removed. In the multiple-choice modality on the basic tier (hatched bars in Figure 2A), basic RAG was already a top-performing strategy, achieving accuracies of up to 97.2%. However, the superiority of RAG became most pronounced in the more challenging open-ended modality. Across both tiers (solid bars in Figures 2A and B), all RAG-based methods—basic, advanced, and hybrid RAG—consistently and significantly outperformed their non-RAG counterparts. For example, in the advanced open-ended setting (Figure 2B), Claude Opus 4’s accuracy increased substantially from 63.6% with RoT to 89.3% with advanced RAG and further to 89.7% with hybrid RAG, representing one of the most substantial performance gains observed in this setting. This underscores that for realistic, open-ended tasks, providing precisely aligned external knowledge is a highly effective strategy for improving accuracy.

However, our results revealed that the effectiveness of specialized RAG is highly dependent on the alignment between the retrieved context and the question’s knowledge domain. This dependency was clearly illustrated by the models’ performance when presented with out-of-domain context. In the advanced multiple-choice setting (hatched bars in Figure 2B), a notable adverse effect was observed for the Claude Opus 4 model, whose accuracy declined significantly to 72.8% when using the out-of-domain basic RAG strategy. A converse pattern was observed in the basic multiple-choice setting (hatched bars in Figure 2A), where the introduction of out-of-domain advanced RAG context also led to a performance drop for some models, such as GPT-4o, declining to 63.2%.

The introduction of the hybrid RAG strategy directly addresses this domain alignment challenge. By having access to both knowledge bases, hybrid RAG simulates a more practical scenario. The results demonstrate its remarkable robustness. In the basic setting (Figure 2A), hybrid RAG’s performance nearly matched that of the specialized basic RAG (eg, 95.2% vs 95.6% for GPT-4o in the multiple-choice modality). Similarly, in the advanced setting (Figure 2B), it remained highly competitive with the top-performing advanced RAG. This indicates that the hybrid RAG approach successfully navigates the knowledge selection challenge, maintaining high accuracy without requiring prior knowledge of the question’s domain. It effectively mitigates the performance degradation seen with misaligned RAG, positioning it as a more generalizable and practical solution for real-world applications.

### Task-Specific Capability Profile: Uncovering True Failure Modes

To further dissect the LLM performance, we analyzed its accuracy across different clinical task categories (Figure S1 in Multimedia Appendix 1). The “scaffolding effect” of selection options was immediately apparent. A visual comparison between the multiple-choice modality (Figure S1A in Multimedia Appendix 1) and the open-ended modality (Figure S1B in Multimedia Appendix 1) reveals a universal degradation of performance across nearly all tasks and LLMs. The radar plots in the open-ended setting are visibly “shrunken,” indicating that the performance drop observed in the overall accuracy is a pervasive phenomenon affecting all facets of clinical tasks.

Our task-specific analysis provides a granular capability profile of current LLMs, offering actionable insights for both biomedical researchers and clinicians.

For researchers, LLMs demonstrated considerable reliability on fact-retrieval–oriented tasks, such as those in the “Molecular and Genetic Basis” category (Figure S1A in Multimedia Appendix 1). This suggests their potential as powerful hypothesis generation assistants. For example, a researcher could rapidly query an LLM about the known molecular features of MSI tumors across different cancer types, accelerating literature review and experimental design.

For clinicians, the profile reveals a critical dichotomy. On one hand, LLMs can serve as reliable and rapid knowledge retrieval tools for well-established facts, such as those in “Diagnosis and Testing.” On the other hand, we pinpointed areas where clinicians must exercise extreme caution. A consistent pattern of underperformance was observed in complex, multifactor decision-making tasks, with “Therapeutic Response (Chemotherapy)” emerging as a prominent failure mode across all models in the challenging open-ended setting (Figure S1B in Multimedia Appendix 1). For instance, under the vanilla prompt, the accuracy of GPT-4o on this subtask (n=21) was only 33.3%, a performance level statistically indistinguishable from random chance in a high-stakes context. This striking weakness indicates that when faced with nuanced clinical scenarios that require weighing competing evidence, the internal knowledge of general-purpose LLMs is dangerously unreliable. This is the domain where unassisted LLM usage poses the greatest risk.

Crucially, our analysis also validates a clear pathway to mitigate these risks through a well-designed RAG system. However, the journey to effective RAG is nuanced. The erratic performance of basic RAG, which sourced knowledge only from general guidelines, highlights the danger of grounding models in incomplete or overly broad information; in some advanced tasks, it even underperformed non-RAG methods. In contrast, advanced-RAG, which used our specialized MSICKB knowledge base, consistently improved performance across most high-risk tasks. The most robust results were achieved with hybrid RAG, which integrated both knowledge sources, leading to substantial and stable performance gains, particularly in the most challenging decision-making areas such as chemotherapy response. This improvement was statistically significant for hybrid RAG with both GPT-4o (*P*<.001; McNemar test) and Gemini 2.5 Pro (*P*=.02) compared to the vanilla strategy, although not for Claude Opus 4 (*P*=.22), reflecting its unique interaction with RAG systems.

This finding provides a clear directive: for high-stakes clinical decision support, RAG is not a monolithic solution but a framework whose effectiveness is contingent upon the quality and comprehensiveness of its knowledge base. A well-curated, specialized knowledge source is an essential safety and performance mechanism.

### AI Safety Analysis: RAG as a Key Enabler for LLM Honesty

**Figure 3.**
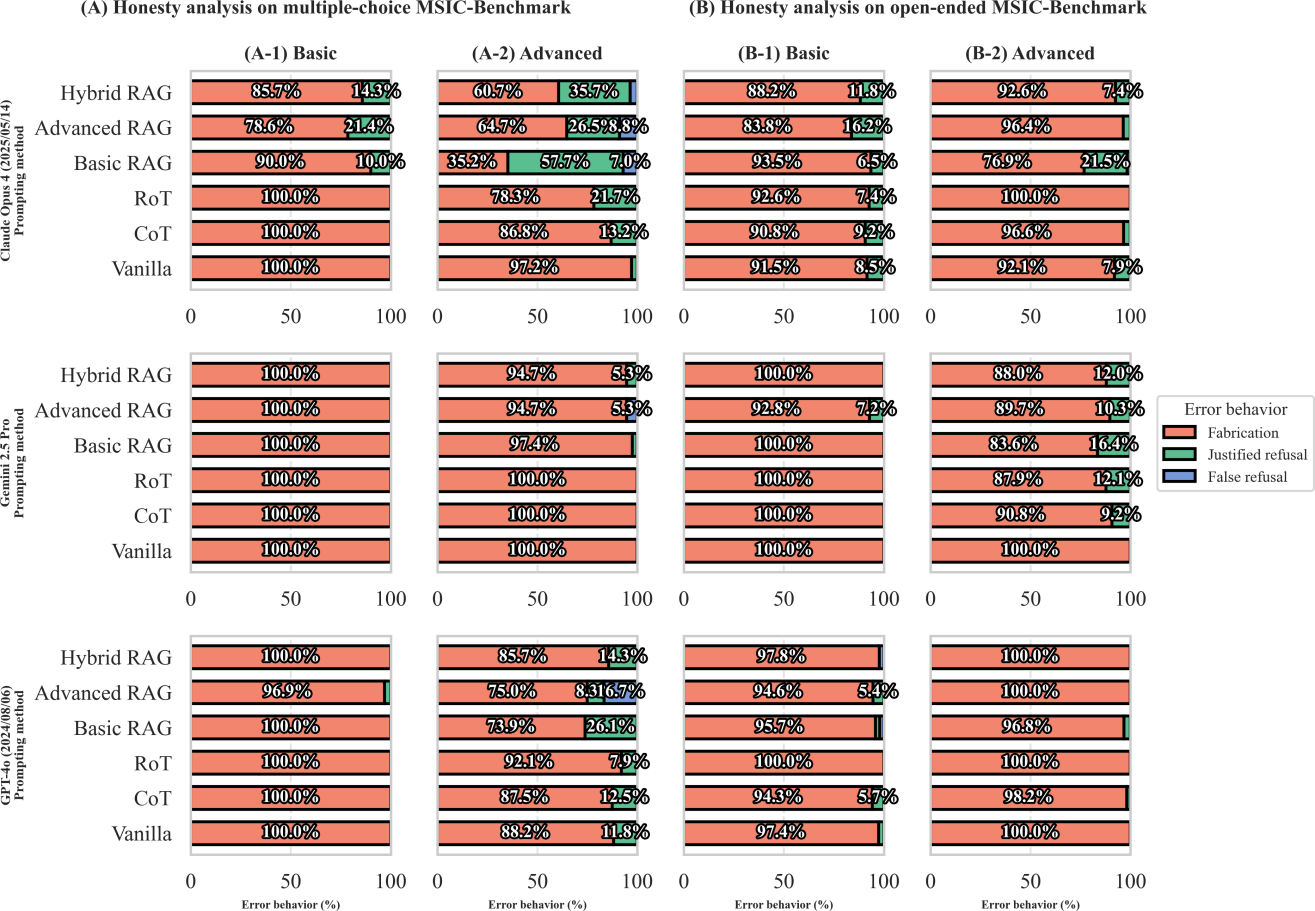
Honesty analysis of error behaviors.

Moving from performance to safety, we analyzed the composition of incorrect responses by categorizing them into 3 distinct behaviors: fabrication, justified refusal, and false refusal (Figure 3). Our analysis begins with the basic tier (Figures 3A and 3B), where a stark pattern emerged: nearly all incorrect responses were classified as fabrication. This outcome, however, is not an indicator of inherent model dishonesty but a direct consequence of the benchmark’s design. The selection-based and true or false questions in this tier did not include a “don’t know” option, structurally forcing the LLM to guess when faced with uncertainty and precluding any form of refusal.

The models’ true behavioral tendencies are revealed in the advanced tier (Figures 3A and 3B), where expressing uncertainty is possible. Even here, non-RAG strategies (vanilla, CoT, and RoT) demonstrate a strong inclination toward fabrication, with rates often exceeding 85%. This highlights a critical safety concern, that is, without a bounded context, LLMs tend to guess rather than admit ignorance. In contrast, the introduction of RAG strategies fundamentally alters this behavior. Across all models and modalities, RAG dramatically reduces the proportion of fabrication by converting these high-risk errors into justified refusals. This shift represents a significant enhancement in model honesty, as the LLMs learn to correctly identify when the provided context is insufficient to form an answer.

However, this safety improvement comes at a price: the emergence of “false refusals,” a failure mode unique to RAG where the model refuses to answer despite the context containing the necessary information. This dynamic is most vividly illustrated by Claude Opus 4 under the basic RAG strategy in the multiple-choice setting (Figure 3A). Here, fabrication plummeted from 97.2% (vanilla) to just 35.2%. This reduction was primarily achieved by converting a remarkable 57.7% of errors into desirable justified refusals. The cost, however, was a 7% rate of false refusals. This pattern—substituting dangerous fabrications with mostly safe justified refusals at the expense of some loss in utility—is a consistent and critical finding of our RAG analysis.

This analysis demonstrates that RAG is a powerful mechanism for inducing epistemic caution. Grounding the model in a bounded context provides a clear basis for assessing knowledge limits, which is the mechanism behind justified refusals. While the existence of false refusals highlights a persistent challenge in ensuring models robustly use all provided evidence, the net effect represents a highly favorable shift in the error profile. RAG systematically replaces high-severity, factually incorrect errors (fabrication) with manageable, lower-severity utility errors (false refusals), thereby making the LLM’s failure modes safer and more trustworthy.

### Error Analysis: Retrieval Failure as the New Bottleneck in RAG System

**Figure 4.**
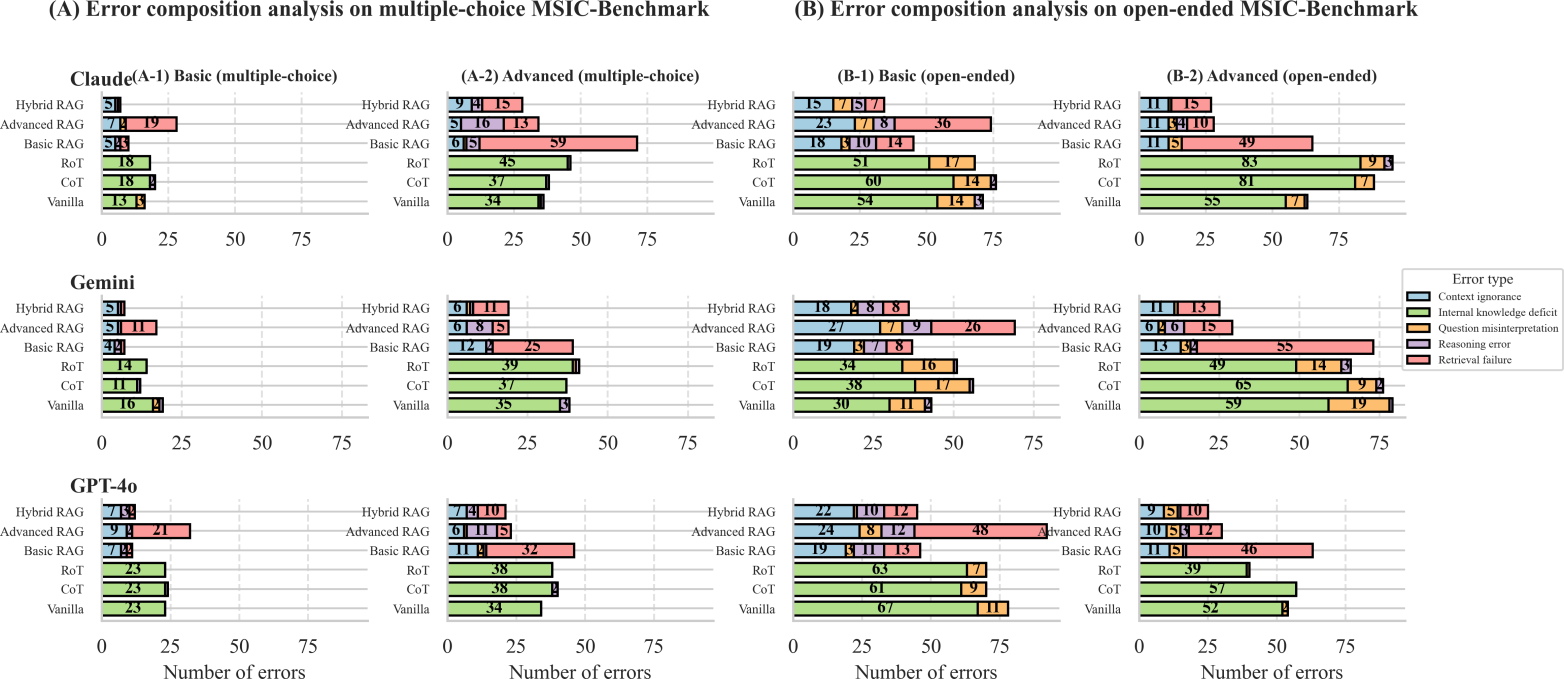
Error composition analysis of incorrect large language model (LLM) responses.

To understand the root causes of errors, we conducted a detailed error composition analysis, applying distinct taxonomies for non-RAG and RAG methods to precisely identify failure points (Figures 4A and 4B).

For methods relying on the LLM’s internal knowledge (vanilla, CoT, and RoT), a clear pattern emerged: failures were overwhelmingly caused by an internal knowledge deficit. This confirms that for this specialized domain, the inherent knowledge gaps of general-purpose LLMs are the primary barrier to accuracy.

The introduction of RAG altered the error distribution. The proportion of internal knowledge deficit errors was substantially reduced across all RAG configurations, as the external knowledge base compensated for the models’ internal deficiencies.

However, this shift revealed that retrieval failure replaced knowledge deficit as the new dominant source of error. In most RAG scenarios, retrieval failure constituted the single largest component of incorrect responses, often accounting for over half of all errors. This indicates that while RAG addresses the problem of “not knowing,” it introduces the challenge of “not finding,” making system performance highly dependent on the accuracy of the retrieval module.

Other RAG-specific errors, such as context ignorance (the LLMs failing to use correct context) and reasoning errors, occurred at a lower frequency. This suggests that when the retriever succeeds in providing the correct context, the LLMs are generally capable of using it appropriately. Therefore, a key area for improving RAG performance in this domain is to enhance the retriever’s ability to consistently deliver the correct context.

### Token Usage Analysis: The High Price of Complex Reasoning and the Efficiency of RAG

**Figure 5.**
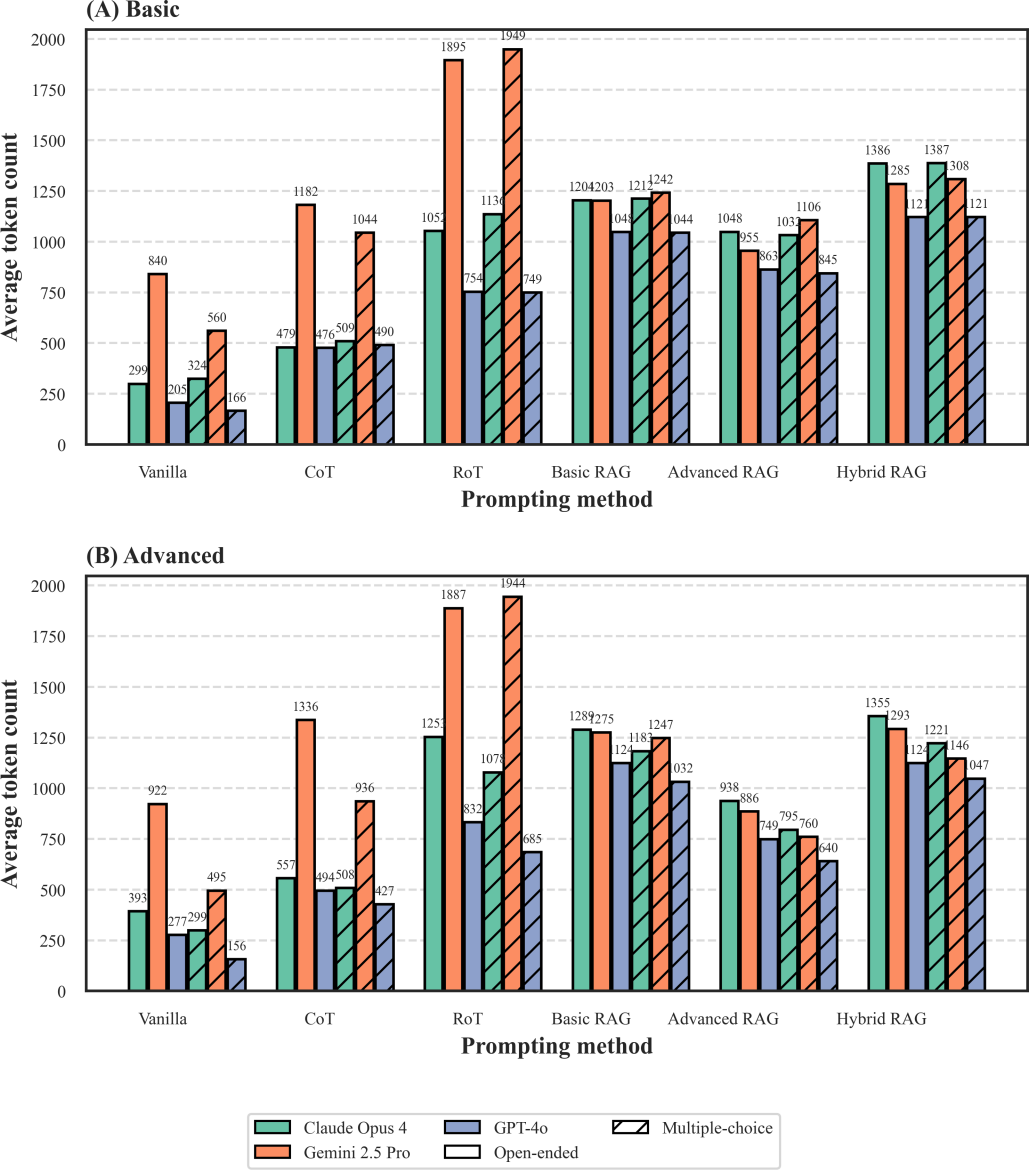
Average total token usage per interaction on the Microsatellite Instability Cancer Benchmark (MSIC-Bench).

Finally, we evaluated the token usage of each strategy by measuring the total token count (input and output) for each interaction (Figures5  6).

The most notable finding, illustrated by the average token counts in Figure 5, was the substantial token usage associated with complex reasoning prompts. The RoT strategy consistently consumed the most tokens across all conditions. This effect was particularly pronounced for Gemini 2.5 Pro in the multiple-choice–Basic setting (Figure 5A), where the average total tokens for RoT (1949 tokens) were nearly 2 times that of CoT (1044 tokens) and almost 4 times that of vanilla (560 tokens). This highlights the significant computational overhead required for multipersona ensemble reasoning.

**Figure 6.**
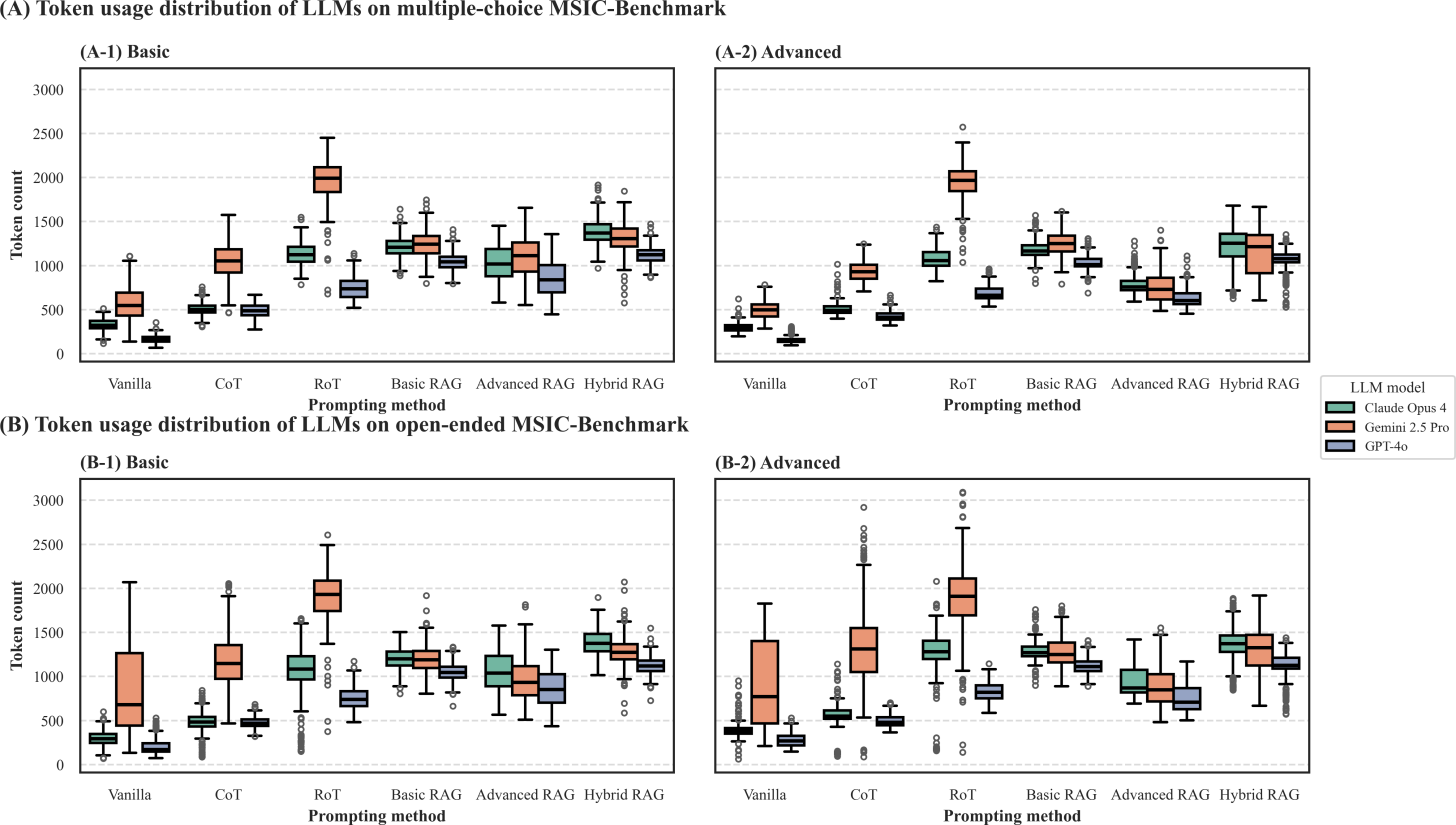
Distribution of total token usage per interaction on the Microsatellite Instability Cancer Benchmark (MSIC-Bench).

The analysis also revealed a clear difference between the 2 evaluation modalities. As shown in both the average counts (Figure 5B) and the distributions (Figure 6B), interactions in the open-ended modality were generally more token-intensive. More importantly, the box plots in Figure 6B show that these open-ended interactions exhibited significantly greater variance (larger IQRs and more outliers) compared to their multiple-choice counterparts (Figure 6A). This indicates that open-ended questions not only require a higher average total token usage but also lead to less predictable and more variable costs.

In terms of efficiency, the family of RAG strategies demonstrated a superior balance of high accuracy and token economy. Crucially, our analysis accounts for the full cost, including retrieved context in the input tokens. While achieving high accuracy (as shown in Figure 2), their average token counts (Figure 5) remained significantly lower than RoT and often comparable to CoT. This highlights a fundamental difference in cost structure: the expense of RoT is primarily in the generated output (ie, computational reasoning), whereas a significant portion of RAG’s cost is front-loaded into the input prompt (ie, providing context). Furthermore, the RAG methods offer a spectrum of cost-performance options: hybrid RAG, which integrates CoT reasoning, logically incurs a higher token cost than basic or advanced RAG, positioning it as a premium option for when maximal accuracy is required. This positions the RAG framework as a strategically efficient approach for deploying reliable clinical AI systems. It allows developers to balance computational cost and performance requirements by selecting the appropriate RAG configuration—from token-frugal methods for simpler tasks to more powerful, context-rich options such as hybrid RAG for high-stakes clinical decisions.

### Performance Analysis Relative to Knowledge Cutoff Date

**Figure 7.**
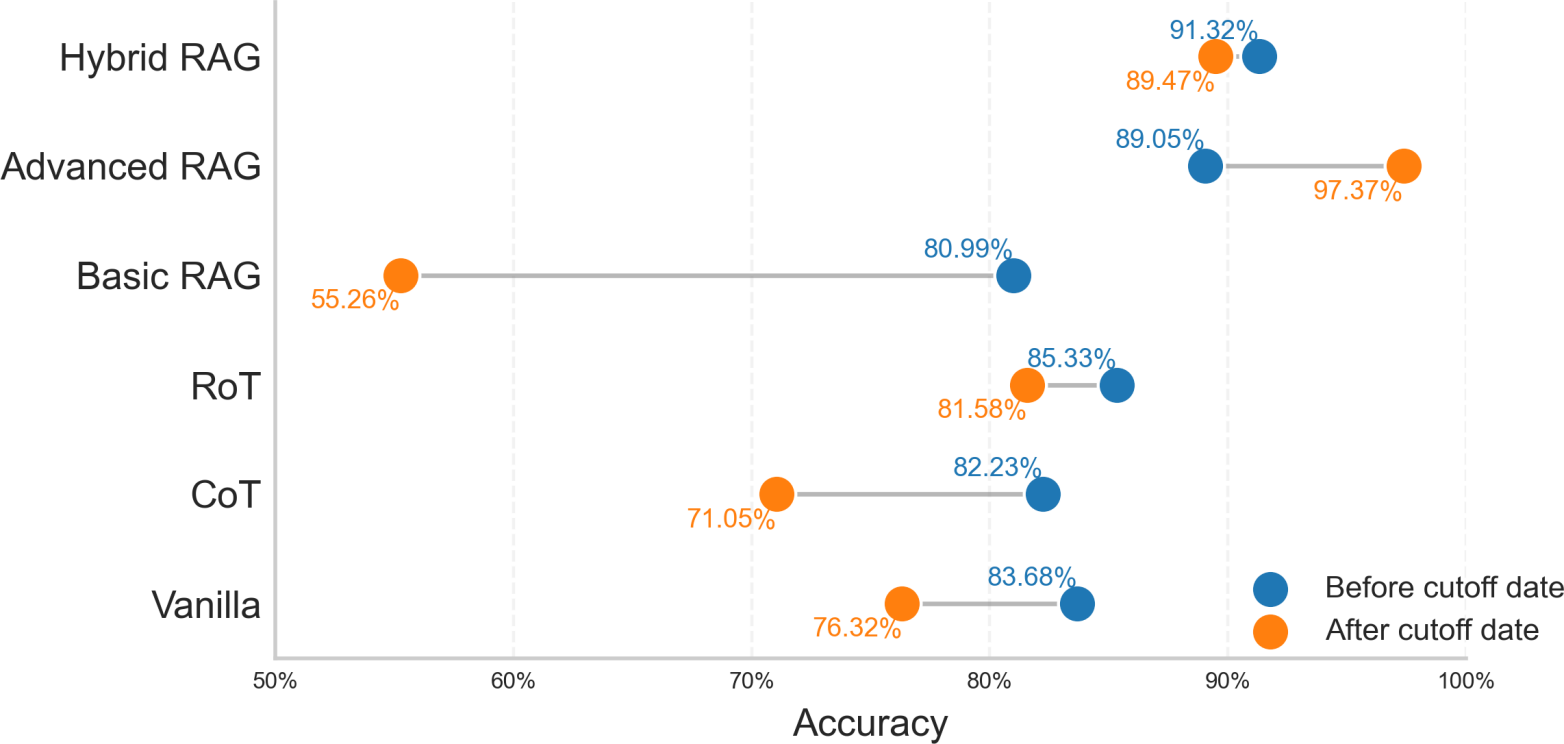
GPT-4o’s (OpenAI) accuracy on questions from before- and aftercutoff sources.

To investigate the influence of training data currency on model performance, we conducted an analysis on the “advanced” tier using GPT-4o, comparing its performance on questions from precutoff vs postcutoff sources (Figure 7).

For strategies relying on internal knowledge (vanilla, CoT, and RoT), performance was consistently higher on the precutoff data (eg, 83.68% vs 76.32% for vanilla). This indicates a dependency on the model’s training history for these non-RAG methods.

The analysis of RAG-based strategies revealed that performance is critically dependent on knowledge source alignment. A striking example is basic-RAG, where providing out-of-domain guideline knowledge to answer advanced questions led to a sharp performance drop on postcutoff data (55.26%), the lowest of any strategy. This highlights the risk that a misaligned RAG can be more detrimental than no RAG at all.

In contrast, when the knowledge source was correctly aligned, RAG’s effectiveness became evident. The advanced RAG strategy achieved the highest accuracy on postcutoff questions (97.37%), demonstrating the model’s strong ability to reason over novel information when the correct context is supplied. Most notably, hybrid RAG maintained high and stable accuracy across both precutoff (91.32%) and postcutoff (89.47%) data, indicating its robustness.

These findings confirm that while non-RAG strategies are sensitive to training data currency, a RAG architecture’s reliability is primarily determined by its knowledge base. A comprehensive and well-aligned knowledge source, as demonstrated by a hybrid RAG, allows the system to perform reliably, independent of the model’s internal knowledge cutoff.

## Discussion

### Principal Findings

The increasing application of LLMs in medicine raises a pivotal question regarding their reliability in knowledge-intensive subspecialties such as MSI cancers, a domain where no gold-standard evaluation benchmark existed at the time of our analysis. To address this challenge, we developed MSIC-Bench. This benchmark features a two-tiered structure based on clinical guidelines (eg, NCCN and ESMO) and a curated, evidence-based knowledge base (MSICKB), allowing for separate assessment of an LLM’s performance on both consensus and nuanced scientific knowledge. Using this framework, we evaluated 3 leading LLMs across 4 prompting strategies, analyzing their accuracy, safety, error composition, and token usage.

Our findings reveal a significant “scaffolding effect,” where selection-based formats inflate accuracy compared to more realistic open-ended scenarios. This performance drop highlighted specific failure modes, notably in chemotherapy-related decisions. To address these failures, RAG proved to be a critical intervention. It not only substantially improves accuracy but also fundamentally shifts the error profile from high-risk “fabrications” to safer “I don’t know” responses. Finally, our analysis highlights key complexities for real-world applications: model-specific vulnerabilities, the importance of knowledge-to-task alignment in RAG, and the high token usage of certain prompting strategies.

Our evaluation of MSIC-Bench yields 3 principal insights. First, we identified a pronounced “scaffolding effect”: LLMs perform substantially better on multiple-choice than on open-ended questions, indicating that widely used multiple-choice benchmarks likely overestimate their real-world clinical capabilities and that honest behavior cannot be assessed unless the task design explicitly allows refusals. Second, error composition analysis shows that in this specialized domain, the dominant failure mode of non-RAG strategies is internal knowledge deficit, meaning that better reasoning prompts alone cannot compensate for missing domain knowledge; with RAG, this bottleneck shifts from “knowing” to “finding,” as retrieval failure becomes the primary source of error. Third, RAG emerges as the key intervention for deploying LLMs in MSI oncology, with performance and safety tightly coupled to knowledge-base quality: domain-aligned and comprehensive resources (as in advanced and hybrid RAG) markedly improve accuracy and convert high-risk fabrications into safer refusals, but also introduce new dependencies on retrieval precision and computational cost, making optimization of the retrieval module and careful curation of knowledge sources (eg, through the use of structured methodologies such as ontologies [35,36] and curated knowledge bases [14,37,38]) central to future clinical AI development.

### Comparison With Prior Work

Our findings are consistent with and extend the growing body of literature on LLM evaluation in specialized domains. For instance, the performance gap we identified between multiple-choice and open-ended modalities (the “scaffolding effect”) aligns with similar discrepancies observed by Song et al [13] in their study on RAG, confirming that evaluation formats significantly impact perceived model capabilities in clinical contexts.

Furthermore, our findings contribute to the continuing discussion about the roles of reasoning vs knowledge in LLM performance. Our conclusion regarding the limited efficacy of reasoning prompts such as CoT in knowledge-intensive tasks is corroborated by prior work. For instance, prior work [39] similarly found that applying CoT in the knowledge-driven domain of rare disease diagnosis could paradoxically decrease accuracy. Our study provides a clear mechanistic explanation for this phenomenon. By using error composition analysis, we demonstrate that when foundational knowledge is absent—as is often the case in specialized medicine—the primary failure mode is internal knowledge deficit. In such scenarios, structured reasoning prompts cannot compensate for the core knowledge void.

Crucially, our work extends this understanding by showing how RAG transforms the problem. We demonstrate that by supplying external knowledge, RAG effectively eliminates internal knowledge deficit, but in doing so, it shifts the primary bottleneck to retrieval failure. This finding refines the distinction between reasoning-intensive and knowledge-intensive tasks, suggesting that for RAG-based systems, retrieval-intensity emerges as a new, critical dimension for analysis and optimization.

### Limitations and Future Directions

Our study has several limitations that also suggest directions for future work. First, MSIC-Bench is limited to a single clinical problem and reflects only a static snapshot of knowledge related to MSI-associated cancers, which restricts its breadth of coverage. Future work should broaden and update the benchmark within MSI cancers by adding more diverse questions, incorporating real-world case-based scenarios, and regularly revising content to reflect evolving evidence and model capabilities. Second, the benchmark is text-only, whereas real-world decision-making is multimodal. An important next step is the development of benchmarks that integrate images, genomic data, and electronic health records. Third, our question-answering format simplifies clinical reasoning, which is often iterative and conversational; future evaluations should include more realistic case-based and dialogue-style tasks.

Fourth, our study used a foundational RAG implementation. Our error analysis identified retrieval failure as the new primary bottleneck, which points to a clear and critical direction for future work. Research should prioritize the investigation of more sophisticated RAG techniques, such as iterative retrieval and query rewriting, to directly address this retrieval challenge. Fifth, our initial RAG evaluation used a “split-index” (oracle routing) strategy. While this design allowed us to isolate the impact of knowledge source quality, we acknowledge that it does not fully reflect real-world scenarios. Our introduction of the hybrid-RAG model was a first step to address this, but future work should focus on developing more sophisticated dynamic routing mechanisms. Sixth, the same static prompt template was applied across all RAG settings. In the advanced and hybrid RAG strategies, the phrase “clinical guideline context” may not have fully matched the nature of the retrieved MSICKB-derived evidence, which could have influenced how the model interpreted the provided context. Future work should explore source-aware prompt designs that better align the instruction wording with the type of retrieved evidence. Seventh, our analysis revealed a critical trade-off between safety and utility in RAG systems. While RAG effectively curbed dangerous “fabrications,” it also introduced “false refusals”—a failure to answer otherwise answerable questions that limits clinical utility. This highlights that the goal for future research is not simply to maximize refusals for safety, but to find an optimal balance. Future work should therefore focus on strategies to minimize false refusals without reintroducing a tendency to fabricate.

Eighth, our analysis of parametric memory was constrained by model cutoff dates, preventing a direct before-and-after comparison for Claude and Gemini. However, our conclusion that “seeing is not knowing” is supported by strong indirect evidence: our error composition analysis (Figure 4) shows that internal knowledge deficit remained the dominant non-RAG failure mode for all models. This finding is consistent with multiple studies showing that LLMs often fail to reliably recall information, even when it is present in their training data, a phenomenon particularly pronounced in specialized, long-tail knowledge domains such as MSI cancers [40,41]. Ninth, as the MSICKB knowledge base and the “advanced” tier questions were developed by the same research group, a discussion of potential circular validation is warranted. To mitigate this risk, every ground truth answer is strictly grounded in and explicitly linked to its source peer-reviewed publication via a PubMed ID. However, more robust external validation remains an important direction for our future work. Our primary planned next step is to collect and annotate real-world clinical data to create an independent test set for validating our benchmark and retrieval framework. We also note that this specific challenge of circular validation, where the retrieval corpus overlaps with the benchmark’s source material, is a widely recognized issue in the evaluation of specialized RAG systems [42,43]. Additionally, our token usage estimates relied on a single tokenizer (tiktoken), which only approximates tokenization behavior for non-OpenAI models. Future work should use model-specific tokenizers to obtain more accurate cost estimates. Finally, because LLMs evolve rapidly, our results capture only a single time point, emphasizing the need for continuous and longitudinal benchmarking in clinical AI.

### Conclusion

In this study, we addressed the critical absence of a standardized evaluation tool for LLMs in MSI cancers by developing and applying MSIC-Bench. Our benchmark revealed that the primary limiter of current LLMs is a profound deficit in domain-specific knowledge. Our results demonstrate that a well-designed RAG architecture is the pivotal intervention to address this knowledge gap. However, it is not a simple fix. We found that RAG transforms the system’s failure modes, shifting the primary bottleneck from knowledge deficits to retrieval failures. This insight establishes a core principle for the development of trustworthy clinical AI: the quality and comprehensiveness of the knowledge base and the precision of the retrieval system are the most critical components. Ultimately, enhancing clinical RAG systems requires a dual focus on optimizing retrieval mechanisms and improving the model’s contextual reasoning. MSIC-Bench provides not only a comprehensive capability profile of LLMs in this domain but also a replicable methodology to guide this future development, ensuring that knowledge-augmented AI assistants can be validated and safely integrated into specialized medical fields.

## Supplementary material

10.2196/88614 Multimedia Appendix 1 Key methodological details—including full system prompts, model specifications, and error analysis taxonomies—to ensure transparency and reproducibility of the main study.

## Abbreviations

AI: artificial intelligence
API: application programming interface
CoT: chain-of-thought
ESMO: European Society for Medical Oncology
LLM: large language model
MSI: microsatellite instability
MSIC-Bench: Microsatellite Instability Cancer Benchmark
MSICKB: Microsatellite Instability Cancer Knowledgebase
NCCN: National Comprehensive Cancer Network
RAG: retrieval-augmented generation
RoT: reflection of thoughts
