# Supplementary material for: Benchmarking Large Language Models and Prompt Engineering Strategies in Microsatellite Instability Cancers: Evaluation Study
Source: J Med Internet Res. 2026 May 21;28:e88614. doi: 10.2196/88614 (PMC13193672; doi:10.2196/88614)
Supplement: Multimedia Appendix 1 [file jmir-v28-e88614-s001.docx]

**
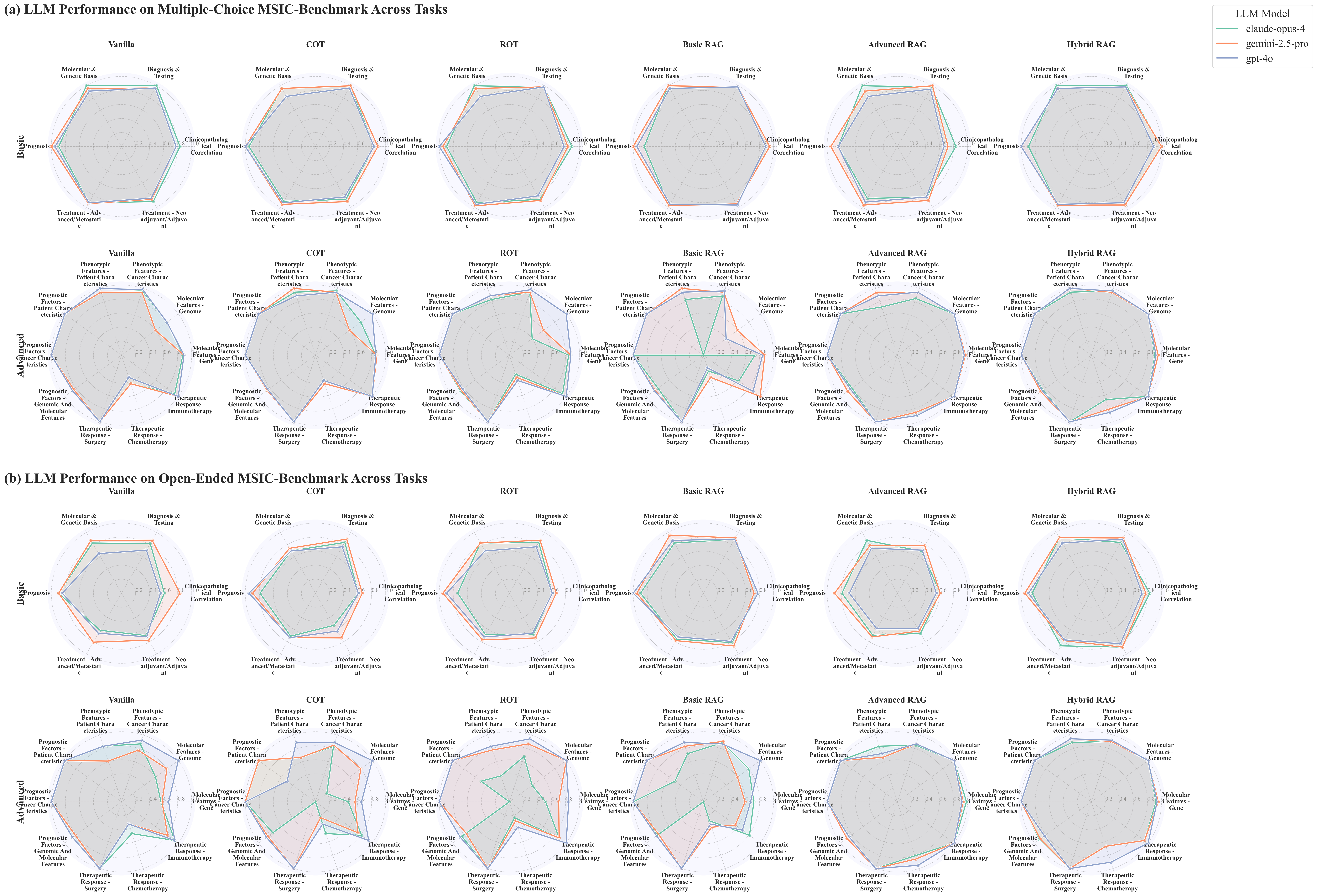
**

**Figure S1**. Task-Specific Performance Profile of LLMs on the MSIC-Bench. The radar charts depict the task-specific accuracy profiles of the three LLMs across the six prompting strategies. (A) Performance on the Multiple-Choice MSIC-Bench. (B) Performance on the Open-Ended MSIC-Bench. Within each panel, the top row of plots corresponds to the Basic tier, and the bottom row corresponds to the Advanced tier. Each axis on the radar plots represents a distinct clinical subtask, with accuracy scaling from 0% at the center to 100% at the outer edge. Certain Advanced-tier subtasks with very small sample sizes (e.g., N ≤ 5) are shown for completeness only and should be interpreted with caution, as their percentage scores are highly volatile and may not reflect stable model performance.

**Section S1:** Full system prompts.

The following are the complete and unabridged prompt templates used in our study for API calls. The {question} and {context} placeholders were dynamically populated with the corresponding data for each query.

PROMPT_VANILLA = """
You are a biomedical expert. Answer the following question based on your internal knowledge.
Question: {question}
"""

PROMPT_COT = """
You are a biomedical expert. Answer the following question. First, provide your step-by-step reasoning process. Then, provide the final answer.
Let's think step by step.
Question: {question}
Reasoning:
[Your step-by-step reasoning here]
Final Answer:
[Your answer here]
"""

PROMPT_ROT = """
You are a biomedical expert. Answer the following question.
Question: {question}
Imagine 3 medical experts are solving this task. Each expert independently provides their step-by-step reasoning and final answer.
After all experts have finished, they discuss together, review and backtrack their previous reasoning steps, and finally reach a consensus on the final answer.
Please present:
[Expert 1's reasoning and answer],
[Expert 2's reasoning and answer],
[Expert 3's reasoning and answer],
[The discussion and the agreed final answer]
"""

The following RAG template was uniformly applied across the Basic, Advanced, and Hybrid RAG settings; only the retrieved context source differed across strategies.

RAG_TEMPLATE = """
You are a biomedical expert. Answer the following question based on the provided clinical guideline context and your internal knowledge.
Question: {question}
Context:
---
{context}
---
"""

**Table S1:** Clinical expert demographics.

| **Expert ID** | **Highest Degree(s)** | **Expert level** | **Years of Post-Graduate Experience** |
| --- | --- | --- | --- |
| Expert 1 | MD, PhD | Senior Clinician | More than 8 years |
| Expert 2 | MD | Junior Clinician | More than 5 years |

This table provides an overview of the anonymized qualifications for the two clinical experts involved in the study's curation and evaluation process.

**Table S2:** Representative examples from the MSIC-Bench benchmark.

| Tier | Task Category | Multiple-choice question | Open-ended question | Reference answer |
| --- | --- | --- | --- | --- |
| Basic (Consensus Knowledge) | Treatment - Neoadjuvant/Adjuvant | For which specific subgroup of patients with stage II dMMR/MSI-H tumors might fluoropyrimidine-based adjuvant therapy be considered?  A. Tumors with poorly differentiated histology  B. Tumors with T3 stage  C. Tumors with T4b stage  D. All low-risk stage II MSI-H tumors " | For which specific subgroup of patients with stage II dMMR/MSI-H tumors might fluoropyrimidine-based adjuvant therapy be considered? | Tumors with T4b stage |
| Advanced (Frontier Evidence) | Molecular Features | In colorectal cancer, how does the gene Mutation Frequency of BRAF V600E differ between the MSI+ tumors group and MSS tumors group?  A. Higher B. Lower C. No significant difference D. Don't know | In colorectal cancer, how does the gene Mutation Frequency of BRAF V600E differ between the MSI+ tumors group and MSS tumors group? | Higher |

This table presents representative examples from the Basic (Consensus Knowledge) and Advanced (Frontier Evidence) tiers of the MSIC-Bench, illustrating the question structure.

**Table S3:** Distribution of questions across tiers, modalities, and subtasks.

| Benchmark | Question Type | Task | Subtask | N (Questions) |
| --- | --- | --- | --- | --- |
| Basic | Multiple choice question | Molecular & Genetic Basis | - | 13 |
|  |  | Clinicopathological Correlation | - | 9 |
|  |  | Diagnosis & Testing | - | 26 |
|  |  | Treatment | Neoadjuvant/Adjuvant | 33 |
|  |  | Treatment | Advanced/Metastatic | 55 |
|  |  | Prognosis | - | 6 |
|  | True or false question | Molecular & Genetic Basis | - | 10 |
|  |  | Clinicopathological Correlation | - | 9 |
|  |  | Diagnosis & Testing | - | 29 |
|  |  | Treatment | Neoadjuvant/Adjuvant | 20 |
|  |  | Treatment | Advanced/Metastatic | 27 |
|  |  | Prognosis | - | 13 |
| Advanced | Multiple choice question | Molecular Features | Gene | 64 |
|  |  |  | Genome | 5 |
|  |  | Phenotypic Features | Cancer characteristics | 53 |
|  |  |  | Patient characteristics | 18 |
|  |  | Therapeutic Response | Immunotherapy | 16 |
|  |  |  | Chemotherapy | 21 |
|  |  |  | Surgery | 1 |
|  |  | Prognostic Factors | Genomic and molecular features | 80 |
|  |  |  | Cancer characteristics | 1 |
|  |  |  | Patient characteristics | 2 |

**Table S4:** Large language model specifications.

| **Model Name** | **API Identifier** | **Knowledge Cutoff Date** | **Official Documentation** |
| --- | --- | --- | --- |
| GPT-4o | gpt-4o-2024-08-06 | 2023-10-01 | [1] |
| Claude Opus 4 | claude-opus-4-20250514 | 2025-03-01 | [2] |
| Gemini 2.5 Pro | gemini-2.5-pro | 2025-01-01 | [3] |

Table S4 provides detailed specifications for the large language models used in this study. "API Identifier" refers to the exact version string used in our experiments to call the model via its API. "Knowledge Cutoff Date" indicates the date up to which the model's training data extends, as reported by the developers. The citations correspond to the official documentation or release notes for each model, as listed in the main manuscript's reference list.

**References**

1. GPT-4o Model | OpenAI API. Available from: https://platform.openai.com/docs/models/gpt-4o [accessed Feb 3, 2026]

2. Claude Developer Platform. Claude API Docs. Available from: https://platform.claude.com/docs/en/release-notes/overview#may-22-2025 [accessed Feb 4, 2026]

3. Model cards. Google DeepMind. Available from: https://deepmind.google/models/model-cards/ [accessed Feb 4, 2026]

**Table S5:** Definitions, categories, and priority order for error composition analysis.

| **Method** | **Error** | **Details** |
| --- | --- | --- |
| Non-RAG | Question Misinterpretation | The LLM misunderstood the intent, scope, or requirements of the question, resulting in an incorrect or irrelevant answer. |
|  | Internal Knowledge Deficit | The question is correctly understood, but the response lacks the key information required for a correct answer. |
|  | Reasoning Error | The question is correctly understood and all key information needed for a correct answer is stated in the response, but the LLM applies the knowledge with flawed reasoning, logic, or calculation, leading to a wrong conclusion. |
| RAG | Question Misinterpretation | The LLM misunderstood the intent, scope, or requirements of the question, resulting in an incorrect or irrelevant answer. |
|  | Retrieval Failure | The question is correctly understood, but the retrieval module fails to retrieve context that contains the key information required to answer the question. |
|  | Context Ignorance | The question is correctly understood, and the retrieved context does contain the necessary key information, but the LLM ignores or fails to use this evidence, resulting in an incorrect answer. |
|  | Reasoning Error | The question is correctly understood, and the retrieved context contains the key information needed to answer the question. The LLM also uses or mentions this information, but its reasoning, logic, or calculation based on this information is flawed, leading to a wrong conclusion. |

This table details the distinct error taxonomies and hierarchical annotation schemes used for Non-RAG and RAG strategies in our error composition analysis.
